# Influence of Diamond Wire Saw Processing Parameters on the Sawn Surface Characteristics of Silicon Nitride Ceramics

**DOI:** 10.3390/mi14091660

**Published:** 2023-08-25

**Authors:** Siyuan Zhang, Yufei Gao, Xingchun Zhang, Yufeng Guo

**Affiliations:** Key Laboratory of High Efficiency and Clean Mechanical Manufacture of MOE, School of Mechanical Engineering, Shandong University, Jinan 250061, China

**Keywords:** silicon nitride ceramics, diamond wire saw slicing, surface morphology, surface roughness, waviness

## Abstract

For the slicing of superhard silicon nitride ceramics, diamond wire sawing technology has great potential for application, and its slicing surface characteristics are an important indicator of cutting quality. In this paper, the sawing experiments of silicon nitride ceramics were carried out within the range of industrial processing parameters of diamond wire sawing (saw wire speed: 800–1600 m/min, workpiece feed speed 0.1–0.4 mm/min). The effects of cutting parameters on the surface morphology, surface roughness and waviness of the as-sawn slices were analyzed. The results show that within the range of sawing parameters for industrial applications, the material on the diamond wire as-sawn surface of silicon nitride ceramics is removed mainly in a brittle mode, with the slice morphology showing brittle pits and regularly distributed wire marks in the 20–55 μm scale range. The surface roughness of the slices along the workpiece feed direction ranges from 0.27 to 0.38 μm and decreases with increasing saw wire speed and decreasing feed rate. The surface waviness ranges from 0.09 to 0.21 μm, which is in good agreement with the changing trend of the sliced-surface roughness. The results of the study provide an experimental reference for promoting the engineering application of diamond wire sawing technology to the processing of silicon nitride ceramic slices.

## 1. Introduction

Silicon nitride ceramics (Si_3_N_4_) have high strength, high hardness, low coefficient of thermal expansion, high thermal conductivity, excellent thermal shock resistance and good oxidation resistance. Si_3_N_4_ is often used in the aerospace and military industry, mechanical engineering, communications, electronics, automotive, energy, chemical and biological fields. It is particularly promising for electronic packaging substrate applications, suitable for applications in complex and extreme environments [1,2,3].

The Si_3_N_4_ ceramics substrate preparation process mainly includes the following procedures: first, the Si_3_N_4_ powder and other mixtures are sintered with Si_3_N_4_ ceramics blocks by using several methods such as atmospheric pressure sintering, hot pressure sintering, reaction sintering, sintering reaction re-sintering, pneumatic pressure sintering, and hot isostatic pressure sintering. Then, after subsequent cutting to obtain ceramic slices, the commercial Si_3_N_4_ substrates are obtained through further grinding and polishing. Currently, the thickness of Si_3_N_4_ ceramics substrates for commercial use ranges from 0.3 to 0.6 mm. Slicing is the forming process of the substrate, which is related to the surface quality of the Si_3_N_4_ ceramics substrate and thus affects the subsequent processing costs. Si_3_N_4_ ceramics slices are mainly processed by wire electrical discharge machining (WEDM) and laser cutting [4,5,6]. WEDM wire cutting of hard-brittle materials has many applications, but when applied to non-conductive Si_3_N_4_ ceramics, requires the addition of an auxiliary electrode, resulting in a complex slicing process [7]. Laser cutting requires high-power lasers for cutting large-thickness workpieces, which limits the application of this technology for cutting large-size Si_3_N_4_ ceramics substrates. In addition, laser cutting causes relatively deep thermal damage layers on the surface and subsurface of the ceramic substrate and slice warpage. In recent years, diamond wire saw slicing technology has been widely used for slicing hard-brittle materials such as photovoltaic and semiconductor silicon crystals [8,9], silicon carbide crystals [10], sapphire [11,12], quartz [13] and magnetic materials [14] due to the advantages of its high sawing performance and small kerf loss. For the slicing processing of Si_3_N_4_ ceramics, the diamond wire saw slicing technology has great application potential.

Surface characterization is one of the most important concerns in machining such as cutting and grinding [15,16,17]. In diamond wire saw slicing, a large number of diamond abrasives on the surface of the diamond saw wire carry out a cutting action to remove material. Scholars have conducted a series of studies on the characteristics of the diamond wire saw processing, focusing mainly on the surface integrity of processing such as slice surface morphology, surface roughness and subsurface damage. The materials processed are mainly focused on silicon crystals. It was found that wire saw cutting parameters have a significant effect on as-sawn slice surface properties [16,17,18,19,20,21]. Yin et al. [16] and Liu et al. [17] found that high wire speeds and low workpiece feed speeds resulted in a better slice-surface quality in their experiments of cutting polysilicon using diamond saw wires with different diameters. Qiu et al. [18,19] found that increasing the saw wire speed and decreasing the feed rate are beneficial for improving the sawing performance and reducing the surface roughness of photovoltaic silicon wafers in multi-wire cutting. Costa et al. [20] found a similar pattern of effects when cutting polysilicon. Huang et al. [21] found that increasing the wire speed can increase the proportion of material ductile domain removal on the slice surface and reduced the slice surface waviness period. Liang et al. [22] analyzed the variation in cutting force during the sawing of monocrystalline silicon, which increased with the increase in workpiece feed and decreased with the increase in wire speed.

Diamond wire saw slicing of Si_3_N_4_ ceramics has great potential for development. Therefore, in this paper, the diamond wire sawing experiments on Si_3_N_4_ ceramics were carried out. The influence of the slicing process parameters (wire speed 800–1600 m/min and workpiece feed speed 0.1–0.4 mm/min) that are practically applied in the industry on the surface properties of as-sawn Si_3_N_4_ ceramics slices was investigated. The study was conducted mainly from the perspectives of slice-surface morphology, surface roughness and waviness. The results of this paper provide an experimental reference for promoting the application of diamond wire saw slicing technology to the processing of Si_3_N_4_ ceramics slices.

## 2. Experiment

### 2.1. Experimental Equipment and Methods

**Figure 1:**
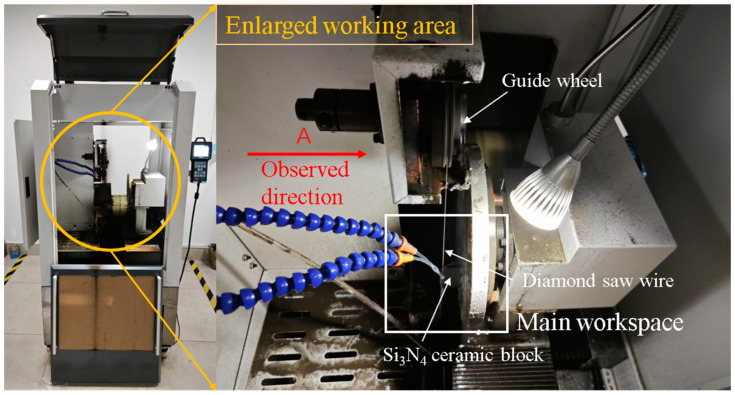
Experimental equipment apparatus.

**Figure 2:**
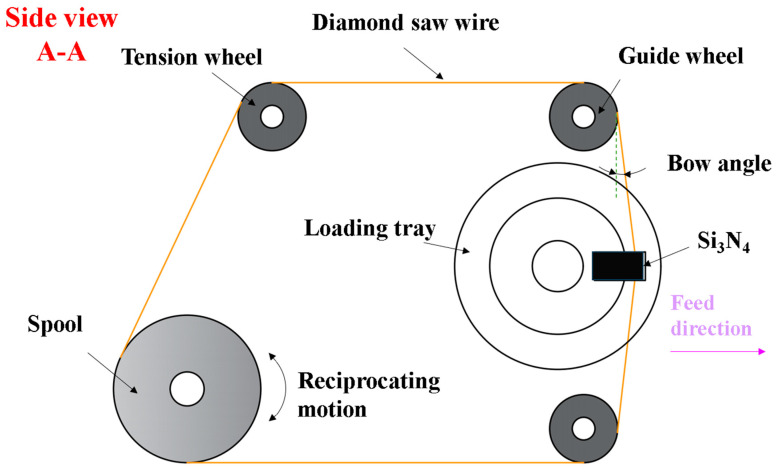
Schematic diagram of the wire saw cutting principle.

The experiment equipment uses a single wire reciprocating cutting machine, which is oriented to cut materials under industrial conditions. The main working part of the machine consists of spools, tensioning wheels, guide wheels, diamond saw wires, loading tray, and cutting fluid spout. The single wire reciprocating cutter maintains a certain tension by means of a tensioning wheel. The diamond saw wire is driven by the spool in reciprocating motion. The workpiece is fixed on the loading tray and driven to provide a feeding motion. Under the combined action of saw wire reciprocating motion and feeding motion, the cutting of Si_3_N_4_ ceramic block in this experiment is completed. Figure 1 and Figure 2 are schematic diagrams of the actual appearance and processing principle of the equipment, respectively.

**Figure 3:**
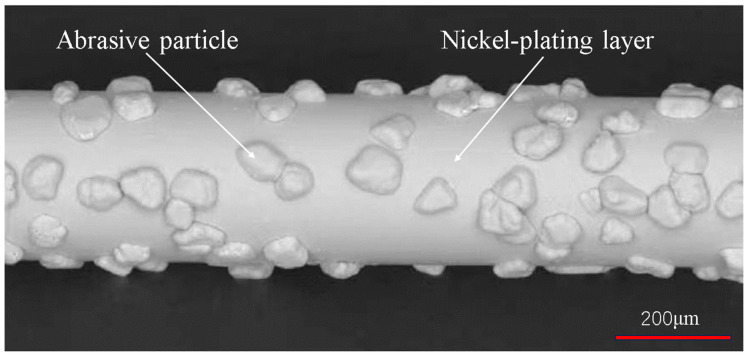
Diamond saw wire appearance.

A 10 mm × 22 mm × 35 mm Si_3_N_4_ ceramic block was selected for this experiment. The workpiece was cut into slices of 1 mm thickness in the direction parallel to the 10 mm × 22 mm surface for detecting the sawn surface quality. The electroplated diamond saw wire was selected and its appearance is shown in Figure 3. The wear state of the processing tool affects the processing characteristics [23,24]. Therefore, the experiments were carried out in the steady state of diamond saw wire wear to avoid the influence of the tool wear on the surface properties of the slices. The parameters of the workpieces and saw wires used in the experiment are shown in Table 1. Pure water is used as cutting coolant.

**Figure 4:**
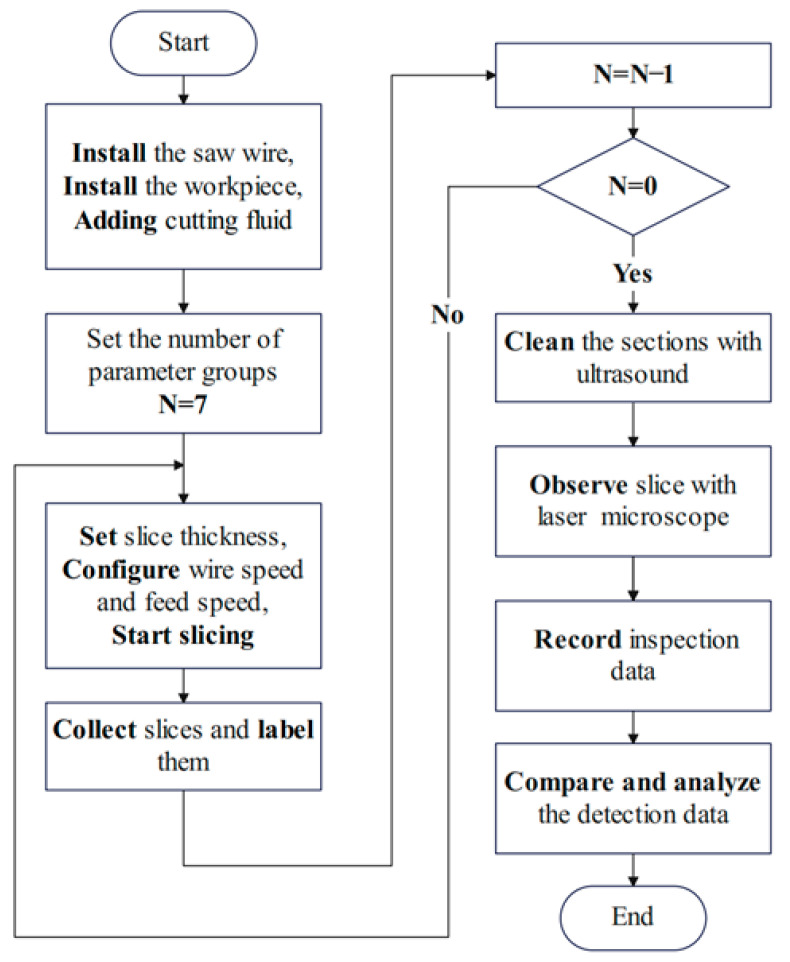
Flowchart of the experiment.

During the experiment, the diamond saw wire is wrapped onto the spool of the device and passes through the correct working position. Afterwards, it is repeatedly tensioned by means of a counterweight. Eventually the diamond wire is evenly wrapped onto the spool with the same degree of tension. The contact surfaces of the specimen and the carrier tray are fixed with two-component epoxy resin adhesive. The cutting experiment starts after checking the status of the cutting machine and the workpiece. The basic procedure from the cutting experiment to the slice quality inspection is as follows (as seen in Figure 4).

Wire speed and feed speed, as two of the important parameters of the wire sawing process, have a large impact on the machining process of hard-brittle materials. Therefore, a single-factor experiment was designed by varying the feed speed and wire speed separately during sawing. As shown in Table 2, a total of seven sets of cutting parameters were adopted for the two sets of experiments. Experiment 1 was set up as four control groups, and the effect of wire speed on the surface quality of as-sawn slices was observed by keeping the feed speed constant. In this case, a control group with a wire speed of 1600 m/min was used to study the machining quality under high speed conditions. Experiment 2 was also set up as four control groups. The wire speed was set as constant and the feed speed as variable. Qualitative analysis and quantitative measurements used the change in feed speed in relation to the change in surface quality.

### 2.2. Evaluation of As-Sawn Surface Characteristics

The evaluation of the as-sawn surface characteristics of Si_3_N_4_ ceramics slices will be approached from both qualitative and quantitative perspectives. The variation laws of as-sawn surface quality with process parameters and the data on the surface quality of the slices will be analyzed, respectively. Surface morphology characteristics, surface roughness, and waviness are important parameters that characterize machining quality [25,26], so these parameters are used to evaluate machining performance in experiments.

**Figure 5:**
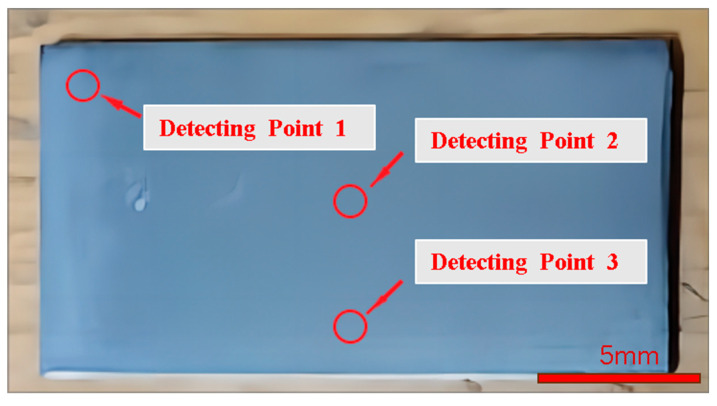
Schematic diagram of sampling detection location.

In order to exclude the effect of random errors on the experimental results, three as-sawn Si_3_N_4_ ceramics slices were cut and labeled under one set of cutting parameters. After ultrasonic cleaning of the slices, three sampling detection positions 1, 2 and 3 were selected as shown in Figure 5. According to the wire sawn morphological characteristics, the roughness value along the specimen feed direction is greater than that along the saw wire movement direction, and the waviness shows a certain pattern of variation along the specimen feed direction. So, the surface roughness and waviness of the Si_3_N_4_ ceramics slices were detected using a KEYENCE Laser Confocal Microscope made in Osaka, Japan along the specimen feed direction. The average value of the sampling data at the three sampling points is taken as the roughness or waviness value of the slice. The average of three slices sawn with the same parameter is taken as the final result. After all the data was collected, the analysis was performed to compare and study the changes in the surface quality of the slices at different wire speeds and feed speeds.

## 3. Results and Discussion

### 3.1. Surface Morphology Characteristics of As-Sawn Slices

**Figure 6:**
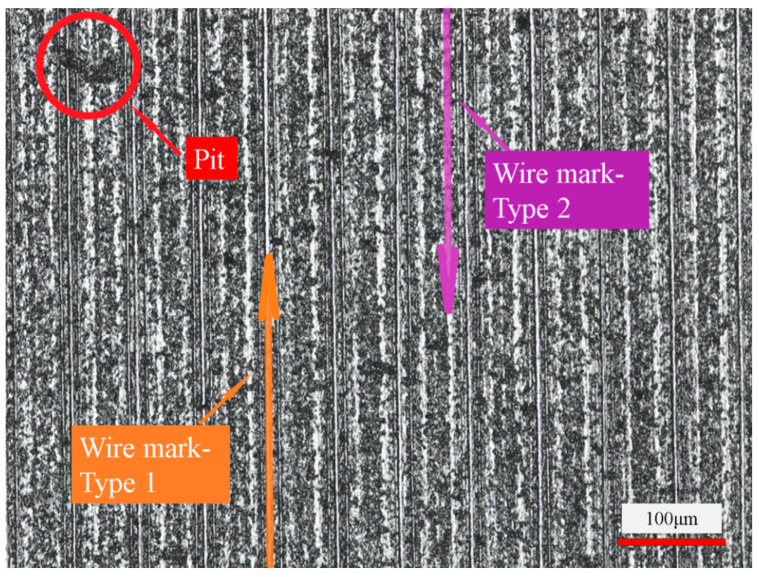
Basic morphology of sliced Si_3_N_4_ surface (wire speed 1000 m/min and feed speed 0.3 mm/min).

**Figure 7:**
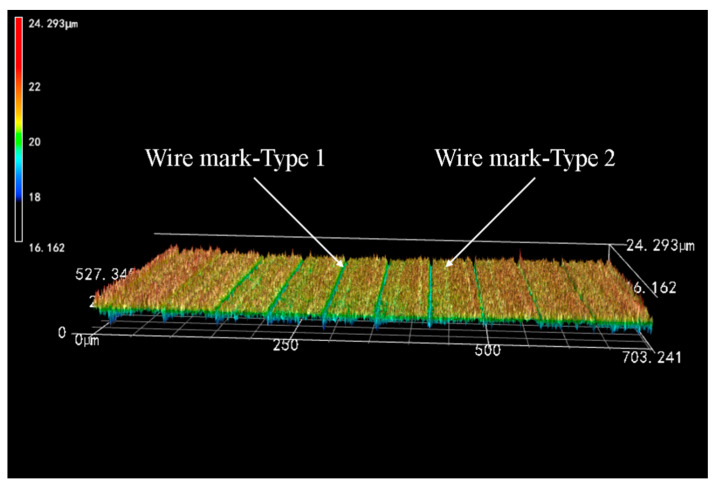
Observation of wire marks on the surface of Si_3_N_4_ slices (wire speed 1000 m/min and feed speed 0.3 mm/min).

Figure 6 and Figure 7 show the surface morphology images of the slices taken using Laser Confocal Microscopy. As can be seen in Figure 6, under this set of process parameters, the formation of the sliced surface is mainly the result of brittle removal of the material, accompanied by a small number of ductile scratches. There are many brittle pits on the surface of the slices, which is due to the material removal form and characteristics of the cutting process of Si_3_N_4_ ceramics. On the one hand, Si_3_N_4_ ceramics are prone to brittle fracture when cutting, and the discontinuous chips result in brittle pits on the machined surface. On the other hand, due to the vibration of the saw wire in cutting [9,17] and the uneven size of the abrasive grains on the diamond saw wire surface [8], then the cut depths of abrasive grains are inconsistent at different positions of the wire surface, which results in an uneven surface. Numerous studies have shown that when the depth of abrasive cutting of hard-brittle materials is below the critical cutting depth, the material can be removed in a ductile manner to obtain high-quality machined surfaces [27,28]. The formation of larger-sized brittle pits left by material brittleness removal affects the serviceability of Si_3_N_4_ ceramics after cutting.

Combined with Figure 6 and Figure 7, the surface of the Si_3_N_4_ceramics slices is also characterized by wire marks of varying depths, which are regularly distributed and periodically varied. Of these, Type 1 wire marks are slimmer in appearance but deeper in depth. The actual depth is basically between 2 and 4 μm, which is in the valley of the overall structural waveform. In contrast, Type 2 wire marks are thicker and more extensive but shallower in depth. The average depth of such wire marks is within 1 μm and is only a slight fluctuation in the overall structural waveform of the surface. Compared with the two types of wire marks, Type 1 wire marks have a greater influence on the surface quality of slices. Therefore, to improve the quality of Si_3_N_4_ ceramics in the wire saw cutting process, the first step is to reduce the depth of the Type 1 wire marks. That is, to reduce the range of height variation in the surface to reduce the degree of waviness and surface roughness.

Combined with the changes in the wire bow angle observed during machining, the cause of the periodic wire marks can be reasonably analyzed. Si_3_N_4_ is a highly hard-brittle material, which is difficult to machine in the production process. When the saw wire touches the workpiece, it is not actually able to quickly remove the excess material and realize the feed at the desired feed rate. As a result, the saw wire bends to create a bow angle. This increases the partial force in the feed direction. When the partial force in the feed direction is small, the wire saw cannot remove the workpiece material in time. It will cut back and forth within a small range of variation, resulting in Type 1 wire marks. When the bow angle increases to a critical point, the wire appears to “jump cut” the situation, along the feed direction to produce a faster jump. On the one hand, the width of the marks increases due to the faster feed. On the other hand, the depth of the marks decreases due to fewer reciprocating cuts in the same position. In this process, Type 2 wire marks appear. The alternation of these two processes during machining produces a periodically changing surface topographic feature.

#### 3.1.1. Influence of Wire Speed on Slice Surface Morphology

**Figure 8:**
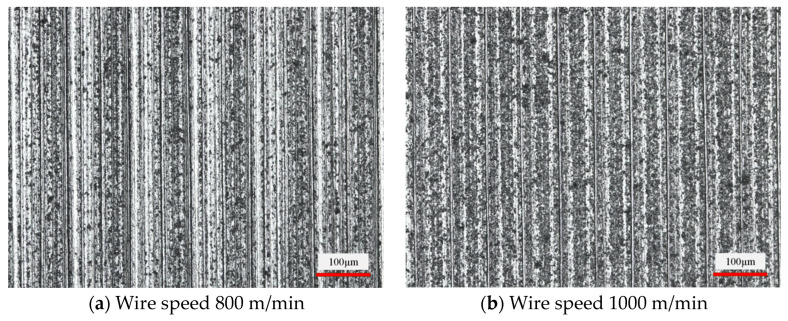

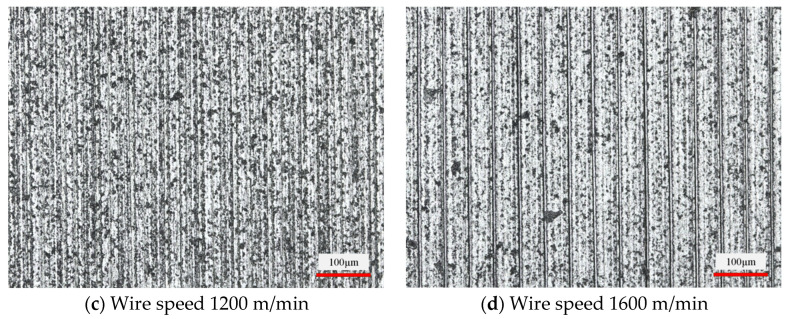
Surface morphology of slices at different wire speeds. (Feed speed 0.3 mm/min).

Figure 8 shows the effect of the diamond wire speed on the slice surface morphology at a feed speed of 0.3 mm/min. With the increase in wire speed, the fluctuation of the surface shape of the workpiece decreases, and the wire marks become clearer gradually. The horizontal distance between the wire marks changes from about 66 μm to 51 μm. The wire marks spacing decreased by 22.7% over the course of the experiment. It is inferred that the density of wire marks is directly proportional to the wire speed in the same size inspection area. This also verifies the assertion made in the previous section. As the wire speed increases, the maximum bow angle produced at the same feed per unit time becomes smaller. The period of surface morphology change in the feed direction is also shortened. From the point of view of machining allowance removal, the change in brittle pits shows a certain trend. The density of brittle pits on the surface of the slices decreased during the increase in wire speed from 800 mm/min to 1600 mm/min. However, the brittle pits did not change significantly in the scale range.

#### 3.1.2. Influence of Feed Speed on Slice Surface Morphology

**Figure 9:**
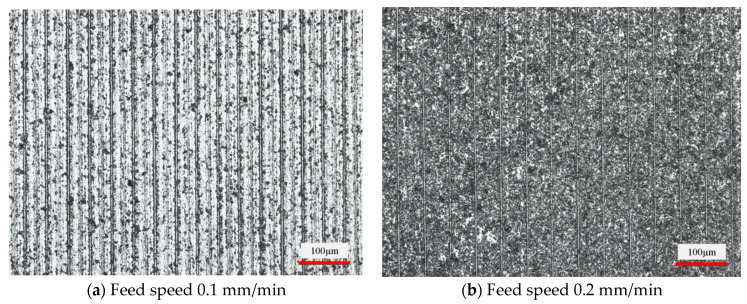

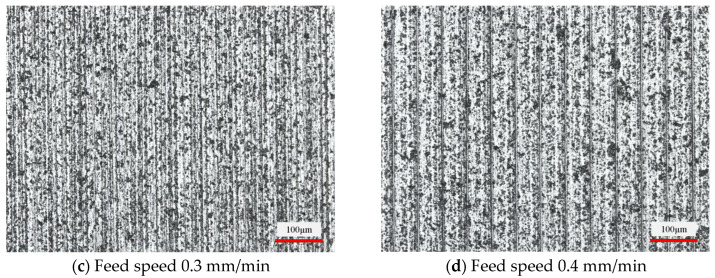
Surface morphology of slices at different feed speeds (wire speed 1200 m/min).

Figure 9 shows the effect of the workpiece feed speed on the slice surface morphology at a wire speed of 1200 m/min. Comparing the difference in surface morphology from Figure 9a–d, it can be observed that the higher the feed speed, the higher the number of brittle pits. The area of a single brittle pit is getting larger and larger. The scale of the brittle pits on the sliced surface varies from 20 to 35 μm to the range of 30 to 55 μm. That is, the surface quality deteriorates as the feed speed increases. An increase in the workpiece feed speed increases the amount of feed per unit of time. The increased proportion of brittle removal in the whole material removal results in more brittle pits. In addition to this, the test process was observed under the condition of constant wire speed. It was found that the diamond saw wire bow angle became larger when the feed speed increased. This exerts a greater feed force on the workpiece, which increases the cut depth of the abrasive grains on the saw wire surface. The size of the pits during material brittle removal also becomes larger.

### 3.2. Surface Roughness R_a_ and Waviness W_a_ of the Sliced Surface

**Figure 10:**
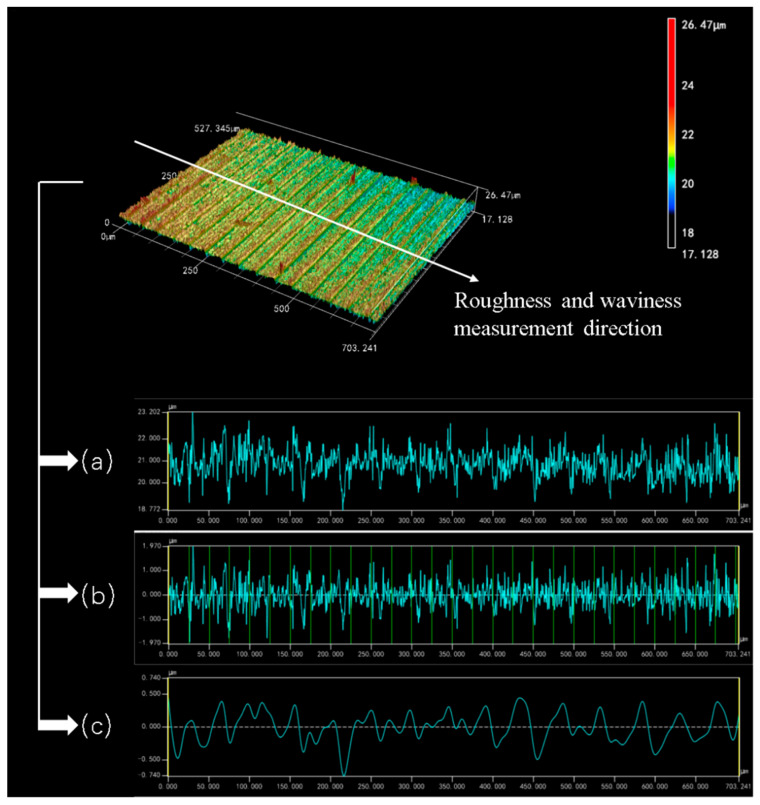
Sliced surface morphology curves; (**a**) measured cross-section graph, (**b**) surface roughness *R*_a_ graph, and (**c**) Waviness *W*_a_ graph. (Wire speed 1000 m/min and feed speed 0.3 mm/min).

Measuring and analyzing the 3D surface images of the Si_3_N_4_ slices, three curves can be derived as shown in Figure 10. Figure 10a exhibits the overall height variation in the sliced surface in the specimen feed direction. Figure 10b,c show the surface roughness and waviness curves of the sliced surface, respectively. The fluctuation of the roughness curves shown in Figure 10b is fairly uniform. A stable roughness value can be obtained over the whole range of the sliced surface. It shows that the diamond wire saw has good processing stability when cutting Si_3_N_4_ ceramics. As can be seen from Figure 10c, the waviness curve on the surface of the Si_3_N_4_ slice shows an irregular curve. The shape of this waviness curve is different from that exhibited when cutting and processing crystalline materials such as silicon crystals and sapphire crystals. According to the results of previous studies, the surface of the cut crystalline material has a sinusoidal-like rippled pattern [8,24]. In contrast, the surface waviness of the Si_3_N_4_ slices in this experiment is more like a high frequency curve similar to irregular vibration. At the two edges of Figure 10c, the difference between neighboring peaks and troughs is between 0.6 and 0.8 μm. While in the middle region of the curve, the difference between adjacent peaks and troughs is around 0.2–0.3 μm. This phenomenon may be related to the material properties of Si_3_N_4_ itself. Si_3_N_4_ is a ceramic-like material made of powder pressed at high temperature and high pressure. The internal microscopic ceramic is prone to uneven density, and even produces local internal stress. The diamond saw wire, as a flexible tool, is easily affected by these factors in the feeding direction. Thus, the microscopic feed trajectory deviates, resulting in this unique surface waviness curve in the Si_3_N_4_ cutting process.

#### 3.2.1. Effect of Wire Speed on Surface Roughness and Waviness of Slices

**Figure 11:**
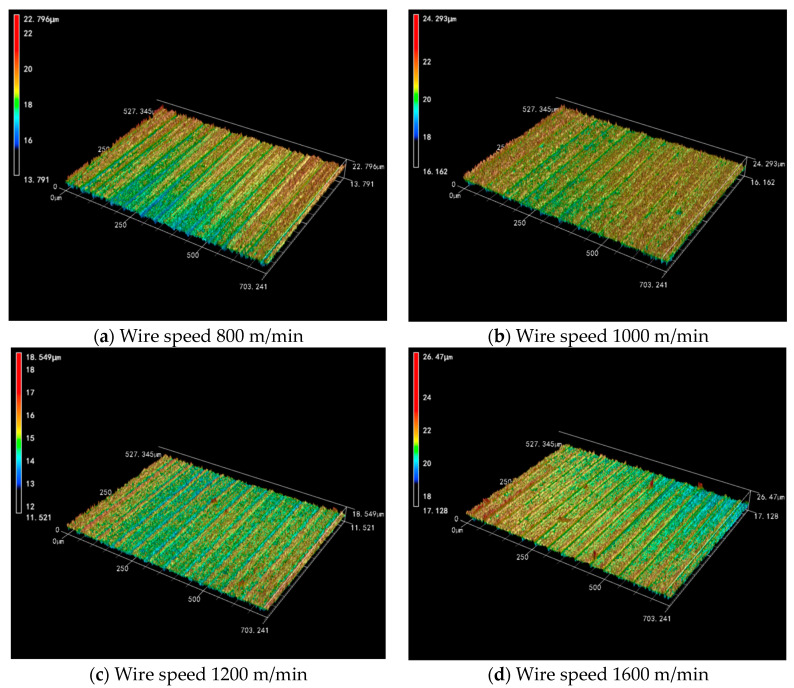
The 3D surface morphology of slices at different wire speeds (feed speed 0.3 mm/min).

The three-dimensional surface morphology can reflect the quality of the machined surface very intuitively. From Figure 11, it can be seen that the surfaces of the Si_3_N_4_ ceramic slices gradually become smooth and flat with the increase in wire speed. Especially from 800 m/mim to 1200 m/min, the variation range of the surface height is 9.005 μm, 8.131 μm and 7.028 μm, which shows that the increase in the wire speed is favorable to improve the surface quality. However, an increase in surface defects occurs locally in the slices during high-speed cutting. Although the cutting ability becomes stronger as the wire speed increases, the vibration amplitude of the wire may also increase. So, under the mutual constraints of two different factors, increasing the wire speed endlessly does not lead to a significant improvement in the surface quality.

**Figure 12:**
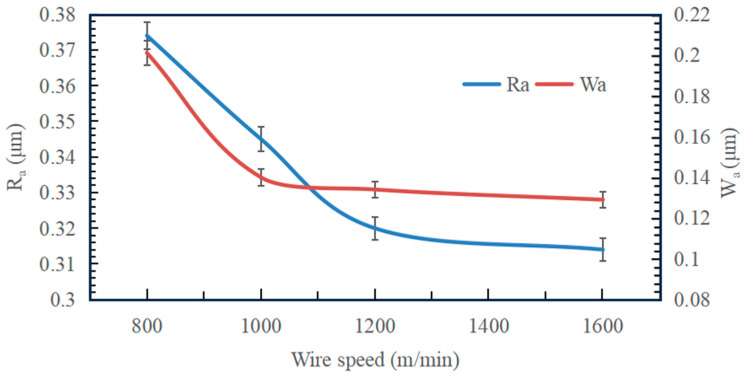
Effect of wire speed on surface roughness and waviness of slices (feed speed 0.3 mm/min).

Figure 12 shows the effect of wire speed on the surface roughness and waviness of Si_3_N_4_ slices at a feed speed of 0.3 mm/min. The surface roughness and waviness of Si_3_N_4_ slices decreased with increasing wire speed. Under four sets of wire speed parameters, the *R*_a_ between neighboring groups decreased by 7.75% and 7.25%, as well as 1.88%. Among them, the decrease in roughness between neighboring groups is more obvious when the wire speed is low. As the wire speed is further increased, especially between the high wire speed of 1600 m/min and 1200 m/min, the value of *R*_a_ decreases insignificantly and finally stabilizes in the range of 0.31–0.32 μm. It can be seen that under low-speed cutting conditions, the change in wire speed has a direct effect on the change in the *R*_a_ value. Under the high-speed cutting condition, the change in wire speed has less effect on *R*_a_. In addition to this, the value of *W*_a_ between neighboring groups decreases by 30.35% and 4.29%, as well as 3.73% when the wire speed increases. The variation in waviness with wire speed also has a similar pattern. However, in the overall curve, it seems that the change in wire speed does not affect the roughness and waviness to a large extent, and the differences between groups are small. Therefore, when processing Si_3_N_4_ ceramics, it is not possible to pursue a too high wire speed, which places higher demands on equipment performance.

#### 3.2.2. Effect of Feed Speed on Surface Roughness and Waviness of Slices

**Figure 13:**
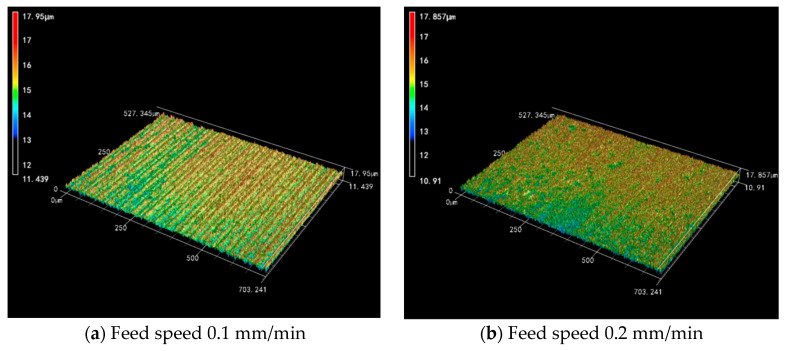

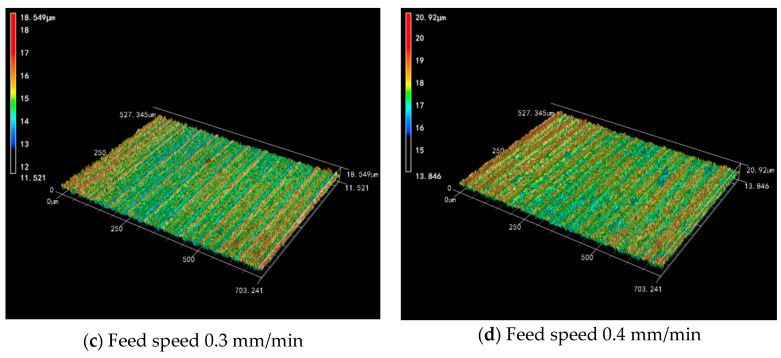
The 3D surface morphology of slices at different feed speeds (wire speed 1200 m/min).

Figure 13 shows the 3D surface morphology images derived from Experiment 2, it can be seen that the height of the Si_3_N_4_ surface keeps changing with the increase in the feed speed. The variation ranges from 6.511 μm, 6.947 μm and 7.028 μm and finally increases to 7.07 μm. It can be learned that the range of height undulation increases with the increase in feed speed. The two have a positive correlation. Compared with the 3D surface morphology images of Experiment 1, the increase in feed speed is more inclined to a uniform decrease in the overall surface quality.

**Figure 14:**
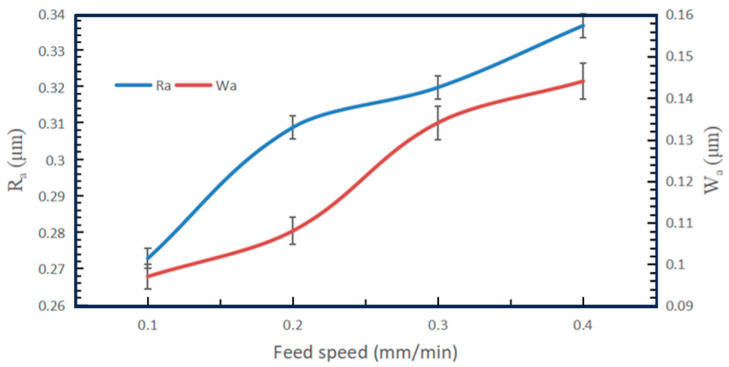
Effect of feed speed on surface roughness and waviness of slices (wire speed 1200 m/min).

Figure 14 shows the effect of the workpiece feed speed on the surface roughness and waviness of Si_3_N_4_ slices at a wire speed of 1200 m/min. With the increase in feed speed, the *R*_a_ and *W*_a_ of the Si_3_N_4_ slices along the feed direction are increasing. Numerically, *R*_a_ between neighboring feed speed control groups increased by 11.65% and 3.44%, as well as 5.04%. The change in *R*_a_ between neighboring groups is proportional to the increase in the feed speed. The variation in *R*_a_ is higher at feed speed values from 0.1 mm/min to 0.2 mm/min. So, the sensitivity of *R*_a_ change is higher at a lower feed speed. When the feed speed is further increased, the sensitivity of the change in the value of *R*_a_ tends to stabilize and increases steadily with the feed speed. From the *W*_a_ curve, it is known that the value of *W*_a_ increases by 10.19%, 19.4% and 6.94% between neighboring groups when the feed speed increases. It can be seen that the variation in *W*_a_ is also positively related to the increase in feed speed and varies more in the range of 0.2–0.3 mm/min. In addition to this, the variation in *W*_a_ is also similar to the variation in *R*_a_.

## 4. Conclusions

This paper carries out an industrial diamond wire saw slicing experiment on Si_3_N_4_ ceramics. The influence law of sawing process parameters used in industrial practical application on the surface morphology, surface roughness and waviness of Si_3_N_4_ ceramic slices were analyzed. The following conclusions are obtained.

Under the sawing processing conditions for industrial applications, the material removal of Si_3_N_4_ ceramics surfaces in diamond wire sawing is predominantly in a brittle mode. The slice morphology shows brittle pits and regularly distributed wire marks in the 20–55 μm scale range.

The surface roughness along the feed direction of the workpiece varied in the range of 0.27–0.38 μm and the surface waviness varied in the range of 0.09–0.21 μm. These two indicators decreased with the increase in wire speed and the decrease in feed speed. The trends of surface roughness and waviness have a good correspondence with the surface morphology of the slices.

## Figures and Tables

**Table 1:** The parameters of saw wire and workpiece.

Parameter	Parameter Value
Workpiece size (mm)	10 × 22 × 35
Slice thickness (mm)	1
Diamond saw wire length (m)	80
Maximum saw wire envelope outer diameter (μm)	350
Type of abrasive grain	Surface nickel plated diamond (25% weight gain)
Abrasive grain size (μm)	30–40
Abrasive grain distribution density (grits/mm)	70–80

**Table 2:** Single-factor experiment table.

Processing Parameters	Wire Speed/m·min^−1^	Feed Speed/mm·min^−1^
Experiment 1	800 1000 1200 1600	0.3
Experiment 2	1200	0.1 0.2 0.3 0.4

## Data Availability

All data generated or analyzed during this study are included in this published article.

## References

[1] Lv X., Huang J., Dong X., Yan Q., Ge C. (2023). Influence of alpha-Si_3_N_4_ coarse powder on densification, microstructure, mechanical properties, and thermal behavior of silicon nitride ceramics. Ceram. Int..

[2] Mir A.H., Ahmad S.N. (2021). A study on fabrication of silicon nitride-based advanced ceramic composite materials via spark plasma sintering. Proc. Inst. Mech. Eng. Part L J. Mater..

[3] Hu F., Zhu T., Xie Z., Liu J. (2021). Effect of composite sintering additives containing non-oxide on mechanical, thermal and dielectric properties of silicon nitride ceramics substrate. Ceram. Int..

[4] Pramanick A., Mandal S., Dey P.P., Das P.K. (2021). WEDM process optimization of sintered structural ceramic sample by using fuzzy-MPCI technique. Mater. Today Proc..

[5] Bharathi V., Anilchandra A.R., Shantanu S.S. (2021). A review on the challenges in machining of ceramics. Mater. Today Proc..

[6] Weixler J., Zweifel M., Schudeleit T., Bambach M., Wegener K. (2023). Laser ablation study of cutting ceramics with consideration of the beam inclination angle. Materials.

[7] Srinivasana V.P., Palanib P.K. (2020). Surface integrity, fatigue performance and dry sliding wear behaviour of Si_3_N_4_–TiN after wire-electro discharge machining. Ceram. Int..

[8] Li X., Gao Y., Yin Y., Wang L., Pu T. (2020). Experiment and theoretical prediction for surface roughness of PV polycrystalline silicon wafer in electroplated diamond wire sawing. J. Manuf. Process..

[9] Liang L., Li S., Lan K., Yu R., Wang J., Zhao W. (2023). Experimental study on the influence of wire-saw wear on cutting force and silicon wafer surface. Micromachines.

[10] Gao Y., Chen Y. (2019). Sawing stress of sic single crystal with void defect in diamond wire saw slicing. Int. J. Adv. Manuf. Technol..

[11] Zhu Z., Gao Y., Zhang X. (2023). Study on subsurface microcrack damage depth of diamond wire as-sawn sapphire crystal wafers. Eng. Fract. Mech..

[12] Wang N., Jiang F., Xu X. (2019). Research on the machinability of A-plane sapphire under diamond wire sawing in different sawing directions. Ceram. Int..

[13] Qiu J. (2023). Fundamental research on machining performance of diamond wire sawing and diamond wire electrical discharge sawing quartz glass. Ceram. Int..

[14] Ban X., Li Y., Han S., Qiu H., Wang X., Cui Z. (2022). Parameters optimization for ferrite slicing based on grey theory. Diam. Abras. Eng..

[15] Yang C., Wang Z., Su H., Fu Y., Zhang N., Ding W. (2023). Numerical analysis and experimental validation of surface roughness and morphology in honing of Inconel 718 nickel-based superalloy. Adv. Manuf..

[16] Yin Y., Gao Y. (2020). Experimental study on slicing photovoltaic polycrystalline silicon with diamond wire saw. Mat. Sci. Semicon. Proc..

[17] Liu Y., Gao Y., Yang C. (2021). Analysis of sawing characteristics of fine diamond wire slicing multicrystalline silicon. Diam. Relat. Mater..

[18] Qiu J., Li X. (2022). Surface formation, morphology, integrity and wire marks in diamond wire slicing of mono-crystalline silicon in the photovoltaic industry. Wear.

[19] Qiu J., Li X. (2020). Formation mechanism of wire bow and its influence on diamond wire saw process and wire cutting capability. Int. J. Mech. Sci..

[20] Costa E.C., Santos C.P., Carvalho V.A., Xavier F.A. (2022). Study on surface integrity and ductile cutting of PV polycrystalline silicon and wear mechanisms of electroplated diamond wire. Int. J. Adv. Manuf. Technol..

[21] Huang H., Zhang Y. (2015). Experimental investigation on the machining characteristics of single-crystal SiC sawing with the fixed diamond wire. Int. J. Adv. Manuf. Technol..

[22] Liang L., Li S., Lan K., Wang J., Yu R. (2023). Fixed-diamond abrasive wire-saw cutting force modeling based on changes in contact arc lengths. Micromachines.

[23] Yang C., Su H., Gao S., Ai Q., Fu Y., Ding W., Xu J. (2021). Characterization and life prediction of single-pass honing tool for fuel injection nozzle. Chin. J. Aeronaut..

[24] Zhu Z., Gao Y., Shi Z., Zhang X. (2023). Study on surface characteristics of as-sawn sapphire crystal wafer considering diamond saw wire wear. Wear.

[25] Liu Q., Cheng J., Liao Z., Luo X., Yang Y., Li M., Yang H., Tan C., Wang G., Ding W. (2023). Research on the light intensity modulation and characterizing methods of surface texture on KDP optics generated in fly-cutting and micro ball-end milling processes. CIRP J. Manuf. Sci. Technol..

[26] Liu Q., Cheng J., Liao Z., Liu M., Chen M., Zhao J., Lei H., Ding W. (2023). Fractal analysis on machined surface morphologies of soft-brittle kdp crystals processed by micro ball-end milling. Materials.

[27] Huang S., Gao S., Huang C., Huang H. (2022). Nanoscale removal mechanisms in abrasive machining of brittle solids. Diam. Abras. Eng..

[28] Li C., Piao Y., Zhang F., Zhang Y., Hu Y., Wang Y. (2023). Understand anisotropy dependence of damage evolution and material removal during nanoscratch of MgF_2_ single crystals. Int. J. Extrem. Manuf..

